# Risk and prognosis of colorectal cancer following bacteraemia with *Streptococcus bovis*–*Streptococcus equinus* complex: A Swedish nationwide retrospective cohort study

**DOI:** 10.1017/S0950268825100836

**Published:** 2025-12-26

**Authors:** Jonas Öberg, Pamela Buchwald, Anton Nilsson, Bo Nilson, Malin Inghammar

**Affiliations:** 1 Department of Clinical Sciences Lund, Section for Infection Medicine, Lund University, Sweden; 2 Department of Infectious Diseases, Helsingborg Hospital, Sweden; 3 Department of Surgery, Skåne University Hospital Malmö, Sweden; 4 Department of Clinical Sciences Malmö, Lund University, Sweden; 5 Department of Laboratory Medicine, Lund University, Lund, Sweden; 6 Department of Laboratory Medicine Lund, Section of Medical Microbiology, Lund University, Lund, Sweden; 7 Department of Clinical Microbiology, Infection Control and Prevention, Office for Medical Services, Region Skåne, Sweden; 8 Department of Infectious Diseases, Skåne University Hospital, Lund, Sweden

**Keywords:** colorectal cancer, infectious endocarditis epidemiology, *S. bovis/S. equinus* complex (SBSEC) bacteraemia, *Streptococcus bovis*

## Abstract

There is a positive association between bacteraemia with *Streptococcus bovis–Streptococcus equinus* complex (SBSEC) and colorectal cancer (CRC). However, the relationship between the timing of SBSEC bacteraemia and CRC is not well-established. Associations with other gastrointestinal cancers have also been suggested. Using national registries, we retrospectively examined the incidence of CRC and other gastrointestinal cancers after SBSEC-bacteraemia in Sweden 2010–2019, and analysed the timing, characteristics, and prognosis of diagnosed CRC. Individuals with SBSEC-bacteraemia were matched to randomly selected controls from the general population at a 1:10 ratio. Cox-regression determined CRC hazard ratios (HR). In total, 908 individuals with SBSEC-bacteraemia were identified and 9,080 controls, of whom 75/908 (8.3%) and 168/9080 (1.9%) respectively had previously diagnosed CRC (*p* < 0.01). During follow-up of individuals *without* previous CRC, CRC was diagnosed in 45/833 (5.4%) individuals with SBSEC and 114/8912 (1.3%) controls (*p* < 0.01). The HR of CRC diagnosis for SBSEC was 10.3 (95% CI 6.7–15.8) overall and 19.8 (95% CI 11.1–35.3) during the first year of follow-up. In conclusion, there was an increased incidence of CRC, and most were diagnosed within the first year. Neither the tumour location, −stage, or -grade of diagnosed CRC nor the rates of other gastrointestinal cancers differed significantly.

## Introduction

Colorectal cancer (CRC) is the third most common cancer worldwide, contributing to the second most cancer deaths [1]. While the incidence is increasing, with age and lifestyle factors being the driving forces, morbidity and mortality have the potential to be reduced through better treatment and CRC screening [2].

In recent years, there has been a growing interest in the association between the microbiome, bacterial infections, and CRC [3–7]. There is a well-known association between *Streptococcus bovis-Streptococcus equinus*-complex (SBSEC) bacteraemia and infective endocarditis, and an increased risk of subsequent CRC diagnosis [3–5, 8, 9]. However, it is unclear whether SBSEC is carcinogenic or just a marker for CRC [8, 10]. Recent epidemiological studies suggest that the increased risk of CRC is primarily attributed to the subspecies *Streptococcus gallolyticus subsp. gallolyticus*, which also causes the majority of SBSEC endocarditis, and experimental studies have shown potential carcinogenic properties [8, 9, 11–15]. The most commonly used method for bacterial identification, matrix-assisted laser desorption/ionization time-of-flight mass spectrometry (MALDI-TOF MS), cannot differentiate between *S. gallolyticus subsp. gallolyticus* and other species when using commercial libraries [16, 17]. Therefore, the association between CRC and SBSEC has mainly been investigated at bacteria group level in register-based epidemiological studies; likewise, subspecies are often undetermined in clinical routine [18]. Extrapolation of the risk of CRC following SBSEC is futile since SBSEC subspecies distribution varies depending on geographical location [9, 19, 20].

For patients with SBSEC bacteraemia, a follow-up colonoscopy to rule out concomitant CRC is often recommended, and some guidelines recommend colonoscopy regularly following infection regardless of findings in the first colonoscopy [8, 10]. However, whether there is an increased risk of developing CRC following the first colonoscopy is currently unknown. In addition, case series have suggested a potential association between SBSEC bacteraemia and other gastrointestinal malignancies, although this has not been thoroughly examined in population-based epidemiological studies [20, 21].

While studies have assessed the CRC incidence among SBSEC patients compared to hospital populations, none have examined the incidence compared to the general population using matched population controls [3–5]. There is still limited data on when CRC is diagnosed in relation to SBSEC bacteraemia and infective endocarditis, and more extensive studies examining a possible association with other gastrointestinal cancers are scarce.

In this nationwide retrospective cohort study, SBSEC bacteraemia and endocarditis in Sweden were investigated in relation to the subsequent risk of diagnosis of CRC and other gastrointestinal malignancies and the characteristics and outcomes of diagnosed CRC.

## Methods

### Study design and population

This study is a nationwide register-based retrospective cohort study of SBSEC-bacteraemia in Sweden 1 January 2010–31 December 2019, linking individual data from national health registries. All individuals registered in Sweden are assigned a personal identity number, enabling cross-referencing with hospital and laboratory databases, as well as national health registries. Sweden is divided into 21 geographic and administrative regions, which are served by 23 clinical microbiology laboratories (Supplementary Figure S1). Data on all episodes of SBSEC-positive blood cultures (SBSEC species and subspecies if available) during the study period were requested from all 23 clinical microbiology laboratories, and 18 delivered the requested data, of which 5 reported limitations (Supplementary Table S1, Supplementary Figure S1). The laboratories delivering data covered approximately 88% of the population in Sweden as of 31 December 2019 [22]. Information on the identification method of the specific isolates was unavailable. Biochemical/phenotypical methods and 16S rRNA gene sequencing were used prior to the introduction of MALDI-TOF MS (year of introduction per laboratory listed in Supplementary Table S1), but details were unavailable.

Inclusion criteria were age ≥ 18 years with a positive blood culture of SBSEC, the occurrence of which defined an episode and its index date in the exposed cohort. Individuals with another positive blood culture after >6 months were included again as relapse episodes for sensitivity analyses. Exclusion criteria were individuals unidentified in the Swedish Total Population Register or those with temporary or re-used personal identity numbers – most often used when immigrating. Exposed episodes were matched 1:10 to randomly selected controls from the general population by Statistics Sweden, based on the year of positive blood culture (index year), geographic region, sex, and year of birth using the Swedish Total Population Register. Controls were all unexposed at the index year and were drawn with replacement. The end of follow-up was 31 December 2021, emigration from Sweden, death, or cross-over to the exposed cohort, whichever occurred first. Date of birth, migration, and death data were collected from the Total Population Register. The underlying cause of death was collected from the National Cause of Death Register.

### Colorectal and other gastrointestinal cancer

Cancer diagnosis data covering the period from 1 January 2000 until the end of follow-up were collected from the National Cancer Register. Detailed information on CRC diagnosed during follow-up was obtained from the Colorectal Cancer Database Sweden (CRCBaSe), covering all rectal cancers in Sweden since 1997 and all colon cancers since 2007.

CRC was defined as in CRCBaSe according to ICD-O/3 topography codes C18-C20 (excluding C18.9) with a histopathology diagnosis of adenocarcinoma, unspecified malign tumour, or unavailable histopathology diagnosis and retrieved from the National Cancer Register. Only the first diagnosis of CRC was recorded. Details on American Society of Anaesthesiologists physical status classification system (ASA) score, CRC clinical stage (tumour, node, and metastasis) location, postoperative histopathology, and oncological outcomes (local recurrence, distant metastasis, and mortality) were retrieved from CRCBaSe.

Colorectal carcinoma in situ was registered in the National Cancer Register according to histopathology diagnosis, with the same topography codes as CRC. ICD-10 codes for benign colorectal tumours from the National Patient Register are listed in Supplementary Table S2.

Other gastrointestinal cancer was defined with topography codes C15-C17 and C21-C26, with a histopathology diagnosis of invasive malignancy with a risk of metastasis, according to the coding in the National Cancer Register.

### Comorbidities and infective endocarditis

Infective endocarditis was registered according to ICD-10 codes on inpatient care during the episode with SBSEC bacteraemia (Supplementary Table S2). Data on colonoscopies from the index date until the end of follow-up and comorbidities from 5 years before until the index date were collected from the National Patient Register, containing diagnoses in inpatient or specialized care, and converted to the Charlson comorbidity index adapted to Swedish ICD-10 lists corresponding to the content of the Charlson/Quan index (Supplementary Table S2) [23, 24]. To improve the capturing of conditions only treated in primary care, data on prescribed drugs for the treatment of diabetes mellitus and chronic pulmonary disease from 1 year before until the index date were obtained from the National Prescribed Drug Register and included in the Charlson comorbidity index (Supplementary Table S2).

### Statistical analysis

Categorical variables were presented as frequency (percent). Associations between categorical variables and exposure status were assessed using the Pearson chi-square test. Continuous variables were presented as median (interquartile range [IQR]). Associations between continuous variables and exposure status were assessed using the Mann–Whitney U-test. Individuals with previous gastrointestinal cancer were excluded in calculations of rates of cancer diagnoses following bacteraemia of the concordant cancer. There were no missing data registered apart from in CRCBaSe, and these were excluded from analyses of data on details on diagnosed CRC.

For the statistical analysis of the CRC follow-up cohort, episodes with previously diagnosed CRC, SBSEC relapse episodes, and those that died within the first 14 days following the index date were excluded. The corresponding unexposed controls were also excluded. Incidence rates and differences of CRC following SBSEC-bacteraemia were calculated [25]. Kaplan–Meier curves for CRC diagnosis following bacteraemia were estimated for the CRC follow-up cohort.

Cox regression was used to estimate the hazard ratios (HR) for CRC diagnosis for individuals with SBSEC-bacteraemia (and subsets of SBSEC species and infective endocarditis), stratified by matching cohorts (matched on index year, geographic region, sex, and year of birth) for the CRC follow-up cohort, with time-on-study as the time-scale. To further control for confounding, we used entropy balancing, a method that resembles propensity score weighting but, unlike the latter, yields perfect covariate balance across cases and controls. The controls were balanced to match the cases with respect to the proportions or averages of the following variables: index year, geographic region, sex, age, and comorbidities (Supplementary Table S2) [23, 26]. The proportional hazards (PH) assumption for the complete CRC follow-up cohort was evaluated graphically by log-minus-log plots and Schoenfeld residuals (results not presented). In the main analyses, the PH assumption was sometimes violated for the exposure variable, meaning that hazard ratios represent weighted averages of time-varying effects [27]. As a complementary analysis, we split the follow-up period into shorter time intervals, within which the PH assumption was never rejected (Schoenfeld residuals test (*p > 0.05)*).

Sensitivity analyses were performed also including relapse episodes. In order to further explore the role of comorbidities and to create an inpatient control group, sensitivity analyses were performed only including SBSEC episodes and controls with a previous hospital stay with a registered diagnosis included in the Charlson comorbidity index [23]. Additional sensitivity analyses for the entropy-balanced model excluded individuals with weights above the 99th percentile or included all controls (in contrast to just the matched controls of the SBSEC-episodes in the follow-up cohort) [28].

A 12-month wash-out period was applied in further analyses to reduce surveillance bias due to the known association between SBSEC and CRC. HRs and Kaplan–Meier curves were estimated from 12 months of follow-up until the end of follow-up for a cohort excluding SBSEC episodes and their corresponding controls with less than 1 year of follow-up due to a diagnosis of colorectal cancer or other reasons for end-of-follow-up.

*P* values <0.05 were considered statistically significant. Statistical analysis was performed using Stata/MP version 18.0 (StataCorp LLC, College Station, TX). Forest plots were created using GraphPad Prism 10.1.2 (GraphPad Software, Boston, Massachusetts, USA).

### Ethics

The study was approved by the Swedish Ethical Review Authority (Dnr 2018/429, Dnr 2020–06313, Dnr 2021–06288-02, Dnr 2022–04728-02), and informed consent was waived in accordance with Swedish regulations. Data were anonymized before analysis.

## Results

### Inclusion, demography, and gastrointestinal cancer

**Figure 1.**
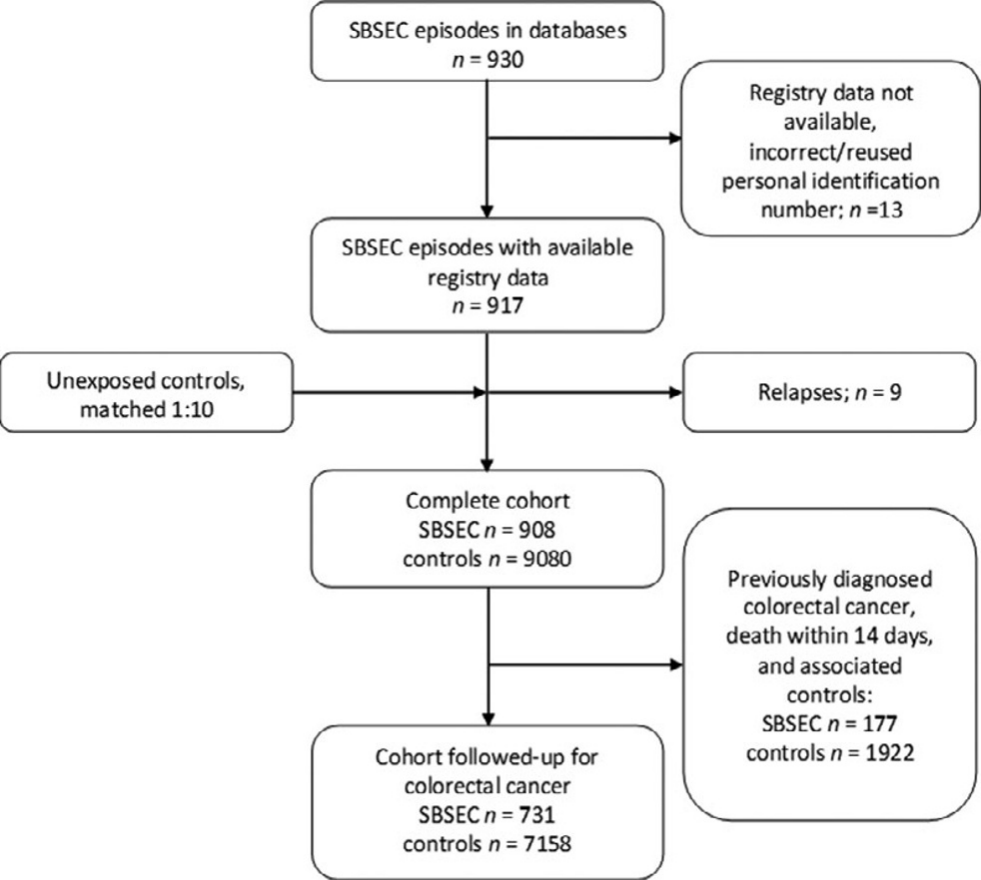
Flow-chart inclusion and cohorts.

The dataset included 930 SBSEC episodes, of which 917 belonged to 908 individuals who were available in the Total Population Register (Figure 1). Demographics of the exposed SBSEC cohort and unexposed controls are described in Table 1.

**Table 1.** Demographics of the complete study cohort

Demographics		Exposed – SBSEC (*n* = 908)	Unexposed controls (*n* = 9,080)
Age, years^a^		77.5 (69.0–85.0)	
Sex, male^a^		5,995 (60.0)	
Charlson index score:	0	110 (12.1)	4,545 (50.1)
	1–2	315 (34.7)	3,179 (35.0)
	3–4	214 (23.6)	930 (10.2)
	≥5	269 (29.6)	426 (4.7)
Diabetes mellitus		284 (31.3)	1,111 (12.2)
Chronic pulmonary disease		207 (22.8)	1,159 (12.8)
Congestive heart failure		245 (27.0)	709 (7.8)
Chronic renal failure		140 (15.4)	304 (3.4)
Chronic liver failure		39 (4.3)	43 (0.5)
Previously diagnosed cancer, all		356 (39.2)	1,477 (16.3)
Previously diagnosed metastasized cancer, all		120 (13.2)	125 (1.4)

*Note:* Categorical variables are presented as *n* (%), ordinal variables are presented as median (interquartile range). There were no missing data.

a Matching variables.

SBSEC species were reported in 450/917 (49.1%) episodes. Of these, 315/450 (70.0%) were reported as *S. gallolyticus* and 135/450 (30.0%) as other SBSEC species. Infective endocarditis was diagnosed in 106/917 (11.6%) of the episodes.

While most underlying gastrointestinal cancers were more prevalent in the SBSEC group than in the unexposed controls, only CRC was diagnosed more often in the SBSEC group following bacteraemia (*p* < 0.01) (Table 2).

**Table 2.** Gastrointestinal cancer of the complete cohort

All gastrointestinal cancer: Previously diagnosed and diagnosed during follow-up of the complete cohort		Exposed – SBSEC (*n* = 908)	Unexposed controls (*n* = 9,080)	*P*
Follow-up total, years		2.1 (0.3–4.1)	4.1 (2.6–6.3)	<0.01
Status at end of follow-up	Deceased	612 (67.4)	2,763 (30.4)	<0.01
	Alive	294 (32.4)	6,258 (68.9)	
	Emigrated	2 (0.2)	55 (0.6)	
	Cross-over to exposed cohort	–	4 (0.0)	
Colorectal cancer	Previous	75 (8.3)	168 (1.9)	<0.01
	Diagnosed	45 (5.4)	114 (1.3)	<0.01
Oesophagus	Previous	2 (0.2)	4 (0.0)	0.04
	Diagnosed	0 (0)	7 (0.1)	0.40
Gastric	Previous	12 (1.3)	9 (0.1)	<0.01
	Diagnosed	1 (0.1)	16 (0.2)	0.65
Hepatic	Previous	4 (0.4)	10 (0.1)	0.01
	Diagnosed	2 (0.2)	18 (0.2)	0.89
Gallbladder, extrahepatic bile ducts	Previous	12 (1.3)	3 (0.0)	<0.01
	Diagnosed	1 (0.1)	9 (0.1)	0.91
Pancreatic	Previous	27 (3.0)	7 (0.1)	<0.01
	Diagnosed	1 (0.1)	17 (0.2)	0.62
Small bowel	Previous	8 (0.9)	6 (0.1)	<0.01
	Diagnosed	1 (0.1)	3 (0.0)	0.27
Anal	Previous	1 (0.1)	4 (0.0)	0.40
	Diagnosed	0 (0.0)	2 (0.0)	0.66
Other gastrointestinal	Previous	0 (0.0)	0 (0.0)	–
	Diagnosed	1 (0.1)	5 (0.1)	0.52

*Note:* Previously known and diagnosed during follow-up of the complete cohort. Individuals with previously diagnosed cancer are excluded from diagnosed cancer during follow-up of the corresponding cancer. Categorical variables are presented as *n* (%), ordinal variables are presented as median (interquartile range). There were no missing data.

### Colorectal cancer

**Figure 2.**
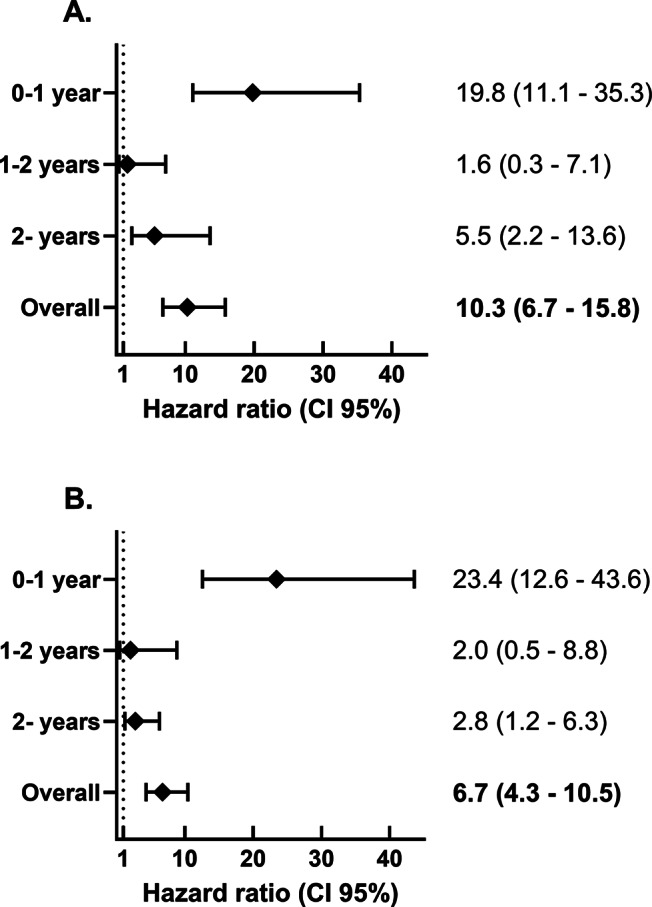
Hazard ratio for colorectal cancer diagnosis following SBSEC-bacteraemia compared to matched controls. (a) Stratified by matching groups. (b) Entropy balancing using the index year, geographic region, sex, age, and comorbidities.

**Figure 3.**
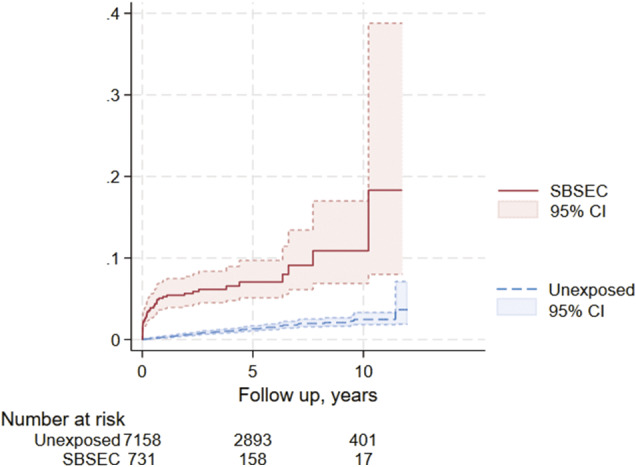
Kaplan–Meier curve of colorectal cancer diagnosis following SBSEC-bacteraemia.

Colonoscopy was performed more often in the SBSEC cohort in the first 12 months after bacteraemia (*p* < 0.01) but not thereafter (*p* = 0.22) (Table 3). Benign colorectal tumours were more common in the SBSEC group (*p* < 0.01) (Table 3). The incidence of CRC after SBSEC bacteraemia was increased overall during follow-up, with HRs of 10.3 (95% confidence interval [95% CI] 6.7–15.8) in the stratified model and 6.7 (95% CI 4.3–10.5) in the entropy balanced model adjusting for comorbidities (Figure 2 and Table 3). The incidence rates of CRCs following SBSEC bacteraemia were most elevated during the first year, while there were no significant differences during the second year, and increased rates were observed again during the remainder of the follow-up (Figures 2 and 3, Table 3). However, most CRCs were diagnosed within the first year, and comparably few cases were diagnosed during the rest of the follow-up. The median time to CRC diagnosis was 0.21 years (IQR 0.02–0.75) in the SBSEC cohort compared to 2.34 years (IQR 1.22–4.41) in the unexposed controls (*p <* 0.01).

**Table 3.** Colorectal cancer diagnosed during follow-up of the colorectal cancer cohort

Colorectal cancer diagnosed during follow-up of the colorectal cancer cohort			Exposed – SBSEC (*n* = 731)	Unexposed controls (*n* = 7,158)	*P*
Colonoscopy		In total	195 (26.7)	437 (6.1)	<0.01
		<12 months	154 (21.1)	108 (1.5)	<0.01
		≥12 months	41 (5.6)	329 (4.6)	0.22
Benign colorectal tumours and polyps^a^		In total	106 (14.5)	187 (2.6)	<0.01
Colorectal cancer	Total	Colorectal cancer, *n*	45	89	
		Incidence per 1,000 person-years	20.4	2.6	<0.01
		Incidence difference (CI 95%)	17.7 (11.7–23.7)		
	Year <1	Colorectal cancer, *n*	35	20	
		Incidence per 1,000 person-years	59.5	2.9	<0.01
		Incidence difference (CI 95%)	56.6 (36.9–76.4)		
	Year ≥1 - <2	Colorectal cancer, *n*	2	20	
		Incidence per 1,000 person-years	4.3	3.1	0.62
		Incidence difference (CI 95%)	1.2 (−4.9–7.2)		
	Year ≥2	Colorectal cancer, *n*	8	49	
		Incidence per 1,000 person-years	6.9	2.4	0.01
		Incidence difference (CI 95%)	4.5 (−0.3–9.4)		

*Note:* Categorical variables are presented as *n* (%).

a Including colorectal adenomas and carcinoma in situ.

When excluding cases and their corresponding controls with less than 1 year of follow-up due to a diagnosis of colorectal cancer or other reasons for end-of-follow-up – and initiating follow-up after 12 months – CRC was diagnosed in 10/506 (1.9%) SBSEC episodes and 31/3697 (0.8%) controls (*p* = 0.02). Only 2/10 (20%) of episodes with SBSEC diagnosed with CRC more than 12 months after SBSEC bacteraemia had undergone colonoscopy within the initial 12-month follow-up period. The HR of CRC among SBSEC episodes following the first 12 months was 4.0 (95% CI 1.8–9.1) in the stratified model and 2.7 (95% CI 1.2–6.2) when using entropy balancing. A Kaplan–Meier curve for the same cohort and period is presented in the supplements (Supplementary Figure S2).

Episodes infected with *S. gallolyticus* species exhibited similar incidence and HRs of CRC as those infected with other SBSEC species overall (Supplementary Figure S3, Supplementary Table S3). Few cases of CRC were diagnosed in those infected with other SBSEC species, especially after the first year following bacteraemia, when the incidence was no longer significantly higher than in the unexposed controls (*p =* 0.08). The highest incidences and HRs were observed in individuals with infective endocarditis during the first year and overall during follow-up in the matched stratified model, whereas these differences were notably less pronounced when adjusting for confounders using entropy balancing (Supplementary Figure S3, Supplementary Table S3).

There were no statistically significant differences in characteristics (tumour location, −stage and -grade) of diagnosed CRC among SBSEC episodes and controls (Table 4). However, the SBSEC episodes diagnosed with CRC had higher ASA and Charlson scores, as well as more local recurrences.

**Table 4.** Details on diagnosed colorectal cancer of the complete cohort

Details on CRC diagnosed during follow-up		Exposed – SBSEC (*n* = 45)	Unexposed controls (*n* = 114)	*P*
Age at diagnosis, years		81.0 (74.7–85.8)	82.1 (76.1–87.2)	0.17
Sex, male		28 (62.2)	75 (65.8)	0.67
Weight, kg^a^		79 (68–87)	75 (68–84)	0.50
BMI^b^		26 (23–28)	25 (23–28)	0.63
ASA-score:	1–2	9 (20.0)	44 (38.6)	0.03
	3–5	20 (44.4)	37 (32.5)	
	Missing	16 (35.6)	33 (29.0)	
Charlson index score^c^:	0	15 (33.3)	64 (56.1)	<0.01
	1–2	17 (37.8)	40 (35.1)	
	3–4	7 (15.6)	7 (6.1)	
	5-	6 (13.3)	2 (2.6)	
Other previous cancer, all^c^ ^,^^d^		14 (31.1)	22 (19.3)	0.11
Other previous metastasized cancer, all^c^ ^,^^d^		1 (2.2)	3 (2.6)	0.89
**Tumour at diagnosis**				
Location:	Colon right	27 (60.0)	47 (41.2)	0.13
	Colon left	13 (28.9)	35 (30.7)	
	Rectum	4 (8.9)	26 (22.8)	
	Multiple	1 (2.2)	2 (1.8)	
	Missing	0 (0)	4 (3.5)	
cT-stage	cT1–2	14 (31.1)	26 (22.8)	0.39
	cT3	11 (24.4)	38 (33.3)	
	cT4	4 (8.9)	16 (14.0)	
	cTX^e^	13 (28.9)	25 (21.9)	
	Missing	3 (6.7)	9 (7.9)	
cN-stage	cN0	21 (46.7)	52 (45.6)	0.37
	cN1–2	11 (24.4)	37 (32.5)	
	cNX^e^	10 (22.2)	16 (14.0)	
	Missing	3 (6.7)	9 (7.9)	
cM-stage	cM0	36 (80.0)	86 (75.4)	0.63
	cM1	7 (15.6)	21 (18.4)	
	cMX^e^	0 (0.0)	0 (0.0)	
	Missing	2 (4.4)	7 (6.1)	
Tumour grade	Low	21 (46.7)	64 (56.1)	0.13
	High	9 (20.0)	13 (11.4)	
	Missing	15 (33.3)	37 (32.5)	
**Follow-up data**				
Surgery primary tumour	Yes	33 (73.3)	86 (75.4)	0.89
	No	9 (20.0)	22 (19.3)	
	Missing	3 (6.7)	6 (5.3)	
Local recurrence	Yes	3 (6.7)	1 (0.9)	0.05
	No	17 (37.8)	45 (39.5)	
	Missing	25 (55.6)	68 (59.7)	
Distant metastasis	Yes	1 (2.2)	10 (8.8)	0.09
	No	19 (42.2)	36 (31.6)	
	Missing	25 (55.6)	68 (59.7)	
Status at end of follow-up	Deceased	24 (53.3)	48 (42.1)	0.20
	Alive	21 (46.7)	66 (57.9)	
	Emigrated	0 (0.0)	0 (0.0)	
CRC underlying cause of death^c^	Yes	13 (54.2)	28 (58.3)	0.74
	No	11 (45.8)	20 (41.7)	
Follow-up, years		1.8 (0.7–3.3)	1.6 (0.5–3.6)	0.91

*Note:* Categorical variables are presented as n (%), ordinal variables are presented as median (interquartile range).

a Missing exposed SBSEC 15; unexposed 34.

b Missing exposed SBSEC 26; unexposed 57.

c No missing data.

d Excluding colorectal cancer.

e Classification incomplete or impossible.

Sensitivity results showed slightly lower HRs when only including the inpatient control group in both analyses, and higher when excluding weights above the 99th percentile when using entropy balancing. These are presented in Supplementary Table S4. Splitting the follow-up time into shorter segments, within which the proportional hazards assumption was never rejected, revealed similar trends to those in the primary analyses shown in Figure 2, with higher HRs during the first year of follow-up and after long-term follow-up (5 years and more). This is displayed in Supplementary Figure S4.

## Discussion

In this nationwide retrospective cohort study, SBSEC bacteraemia was associated with a higher incidence of CRC diagnoses than in the general population, especially within the first year. This increase might partly be attributed to surveillance bias, as many colonoscopies were conducted during this period due to the known association with CRC. However, even after 2 years of follow-up, the incidence rate ratio remained elevated when such bias had likely diminished.

A similar association between SBSEC and gastrointestinal cancers other than CRC has previously been proposed [20, 21]. Our findings revealed that most gastrointestinal cancers were more prevalent among individuals with SBSEC-bacteraemia. However, during the follow-up period, there was no significant increase in the diagnoses of other gastrointestinal cancers following SBSEC bacteraemia compared to the general population, suggesting that such an association of clinical importance is unlikely to exist.

Previous studies have investigated the association between SBSEC bacteraemia and CRC diagnosis using different methodologies. Laupland et al. found a relative risk of 4.4 (95% CI 2.7–6.8) in the first year after infection compared to other bloodstream infections in Queensland, Australia [5]. Kwong et al. in Hong Kong found an HR of 3.8 (95% CI 2.3–6.3) with no increase after 374 days [3]. In Denmark, Justesen et al. reported an HR of 8.5 (95% CI 3.5–20.4) within the first year and 4.4 (95% CI 2.0–9.7) overall using blood culture-negative controls [4]. The studies generally had lower numbers than the present study, which used matched population controls (although adjusted for underlying comorbidities). In contrast to previous studies, we also found increased CRC diagnoses later in the follow-up period. SBSEC has previously been associated with premalignant colonic lesions and early-stage CRC, consistent with findings by Kwong et al. [3, 8]. We found no statistical support for differences in the tumour stage or -grade of diagnosed CRC between the SBSEC cohort and the control group. However, there was a higher frequency of benign colorectal tumours, which may be attributed to increased colonoscopies, as benign colorectal tumours are prevalent in the general population [29].

While this study represents a comparatively large study on SBSEC, essential limitations remain to be acknowledged. Although a slight increase in CRC incidence over follow-up time was observed, the differences were notably reduced when adjusting for comorbidities, and the number of episodes included with long-term follow-up was insufficient to draw definite conclusions. Moreover, commercial and commonly used methods during the study period, such as MALDI-TOF MS and 16 s rDNA, have limitations in identifying SBSEC species and subspecies [16, 17, 30, 31]. Register-based SBSEC studies can, therefore, be expected to have severe limitations in the reliability of reported subspecies. However, these methods can be expected to distinguish *S. gallolyticus* from the other species of SBSEC on species but not subspecies level [16–18, 30]. The association between SBSEC bacteraemia and subsequent CRC diagnosis is most prominent for the subspecies *S. gallolyticus* subsp. *gallolyticus*, while it is still uncertain whether a similar association exists for the other SBSEC species and subspecies [9]. In the present study, *S. gallolyticus* and other SBSEC species had higher rates of diagnosed CRC following bacteraemia than the general population. Still, the number of episodes with defined species was limited, especially for other SBSEC species. *S. gallolyticus* subsp. *pasteurianus* has previously been shown to be the predominant SBSEC- and *S. gallolyticus*-species in Skåne in southern Sweden – a region contributing ¼ of included SBSEC episodes in the present study. However, the distribution of the subspecies in *S. gallolyticus* differs across geographic areas, and is unknown in the rest of Sweden, making it impossible to directly compare CRC rates associated with *S. gallolyticus* bacteraemia in different regions [9, 19, 20]. Since the subspecies *S. gallolyticus* subsp. *gallolyticus* has been found to have the strongest association with CRC and causes most infective endocarditis; the high incidence of CRC following infective endocarditis is likely to be a surrogate marker for bacteraemia with *S. gallolyticus* subsp. *gallolyticus* [8, 9, 15]. Interestingly, the rates of CRC in the SBSEC cohorts with and without infective endocarditis were notably more similar when adjusting for comorbidities. We adjusted models for a wide range of confounders. However, residual confounding from factors such as socioeconomic status, lifestyle, and symptoms prompting blood culturing cannot be excluded.

In conclusion, we found that the incidence of CRC following SBSEC bacteraemia was elevated compared to the general population, especially when infective endocarditis was diagnosed. There was no significant increase in diagnoses of other gastrointestinal cancers following SBSEC bacteraemia, and work-up for non-CRC gastrointestinal cancer due to SBSEC bacteraemia is likely to be futile. While most CRCs were diagnosed within the first 12 months, an increase in CRC incidence rates after 2 years of follow-up could be attributed to potentially carcinogenic properties of SBSEC [11, 12, 14]. However, these diagnoses could just as likely be previously missed CRC, and the differences were reduced when adjusting for comorbidities. Larger studies with longer follow-ups, conducted at the SBSEC subspecies level, are required to determine whether colonoscopies should be performed regularly following SBSEC-bacteraemia.

## Supporting information

10.1017/S0950268825100836.sm001 Öberg et al. supplementary material Öberg et al. supplementary material

## Data Availability

The data supporting this study’s findings are available from the corresponding author upon reasonable request.

## Supplementary material

The supplementary material for this article can be found at http://doi.org/10.1017/S0950268825100836.
