# Supplementary material for: Risk and prognosis of colorectal cancer following bacteraemia with Streptococcus bovis–Streptococcus equinus complex: A Swedish nationwide retrospective cohort study
Source: Epidemiol Infect. 2025 Dec 26;154:e2. doi: 10.1017/S0950268825100836 (PMC12780919; doi:10.1017/S0950268825100836)
Supplement: Öberg et al. supplementary material [file S0950268825100836sup001.docx]

# Epidemiology and Infection

# Risk and prognosis of colorectal cancer following bacteremia with *Streptococcus bovis-Streptococcus equinus*-complex: A Swedish nationwide retrospective cohort study

Jonas Öberg, MD; Pamela Buchwald, MD, PhD; Anton Nilsson, PhD; Bo Nilson, PhD; Malin Inghammar, MD, PhD.

## Supplementary Material


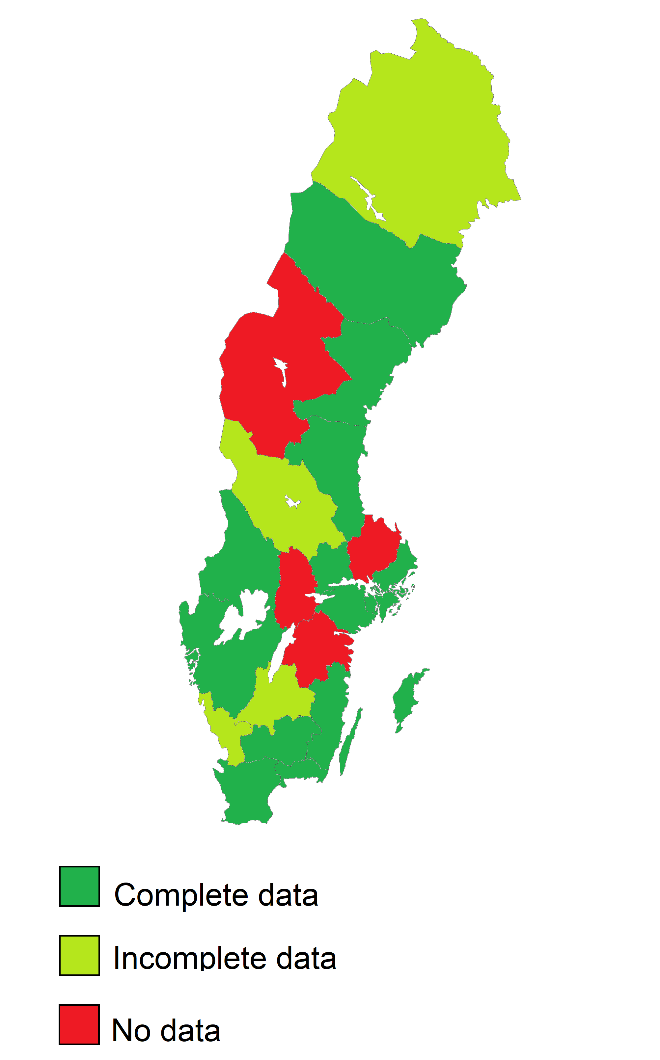


**Figure S1. Geographic regions of Sweden, data coverage.** Dark green – Complete data; light green – Not full coverage; red – Data not received. Source: Statistics Sweden.


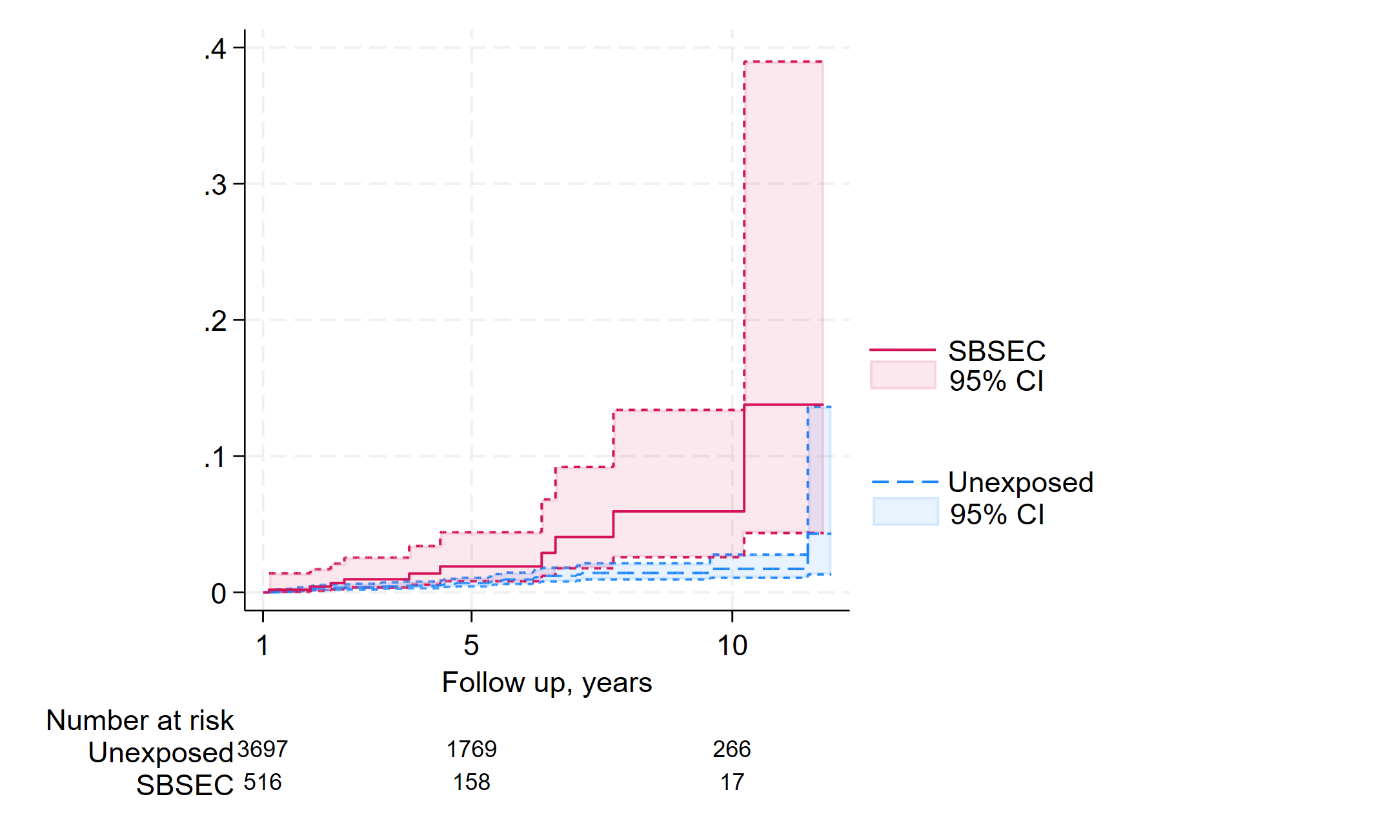


**Figure S2. Kaplan-Meier curve of colorectal cancer diagnosis following SBSEC-bacteremia with follow-up start after 12 months.** SBSEC cases and the corresponding controls with less than one year of follow-up due to a diagnosis of colorectal cancer or other reasons for end-of-follow-up excluded.


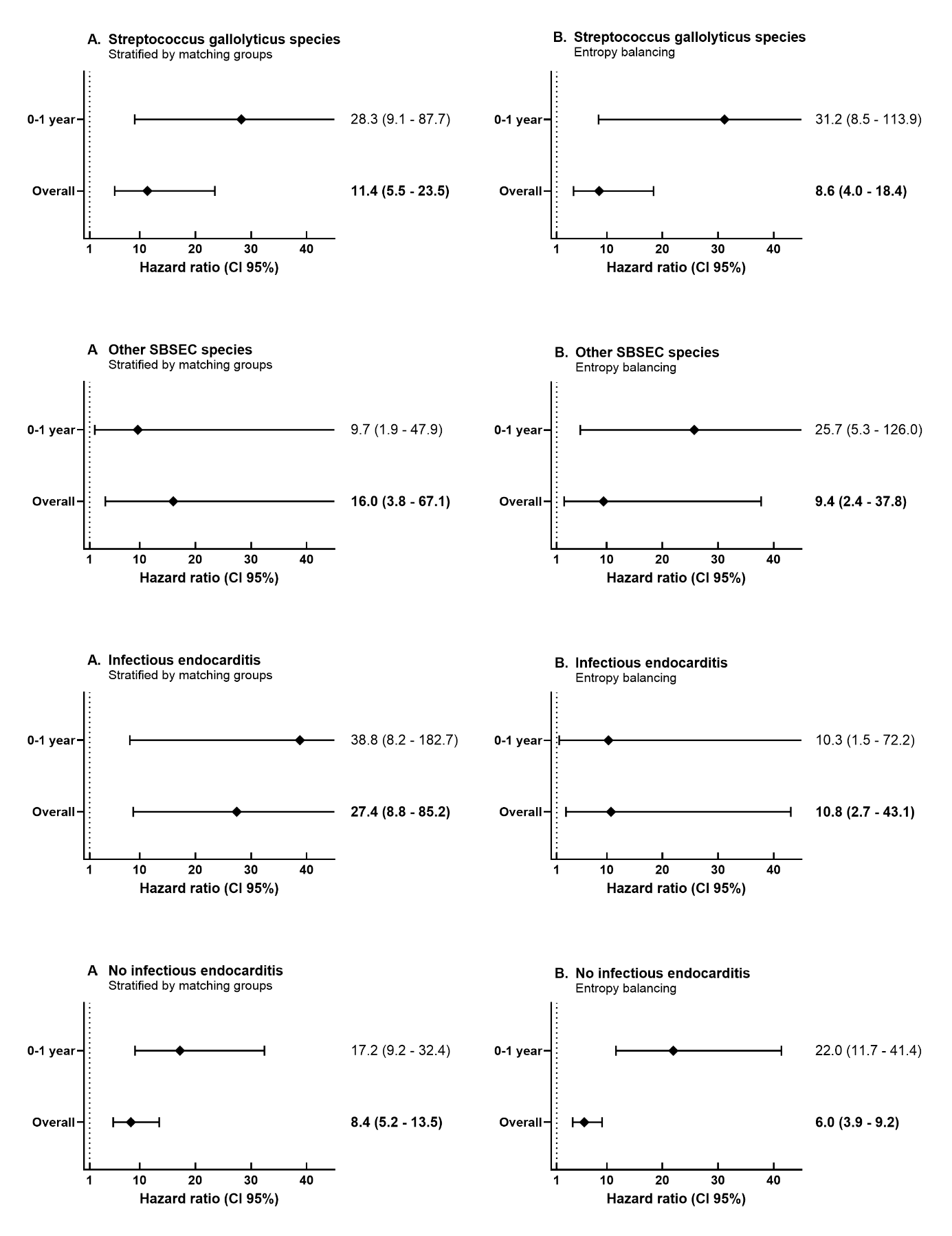


**Figure S3.** **Hazard ratio for colorectal cancer diagnosis following** ***Streptococcus gallolyticus*-bacteremia, other SBSEC-species bacteremia, infectious endocarditis and no infectious endocarditis compared to matched controls.** *Streptococcus gallolyticus* species include *Streptococcus gallolyticus* subsp. *gallolyticus*, *Streptococcus gallolyticus* subsp. *pasteurianus*, and *Streptococcus gallolyticus* subsp. *macedonicus*. Other SBSEC species include *Streptococcus lutetiensis* (previously *Streptococcus infantarius* subsp. *coli*), *Streptococcus infantarius* (previously *Streptococcus infantarius* subsp. *infantarius*), *Streptococcus equinus*, and *Streptococcus alactolyticus*. A. Stratified by matching groups. B. Entropy balancing using the index year, geographic region, sex, age, and comorbidities.

**
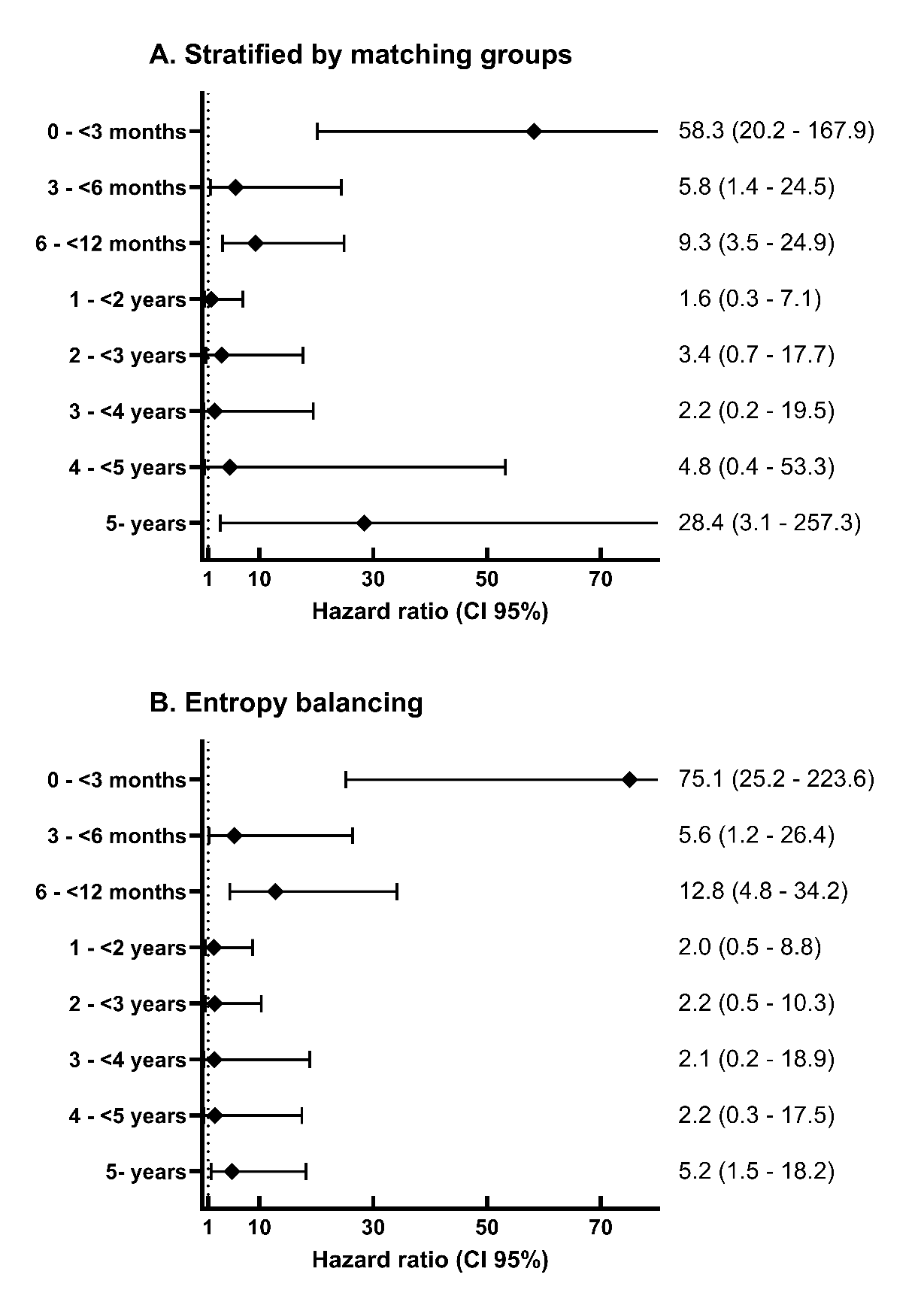
**

**Figure S4.** **Sensitivity analyses of hazard ratios for colorectal cancer diagnosis following SBSEC-bacteremia compared to matched controls.** Split into shorter time periods, each fulfilling the proportional hazards assumption (Schoenfeld residuals test p>0.05).

| **Nr** | **Laboratory** | **Reason for exclusion** | **Episodes reported** | **Introduction of MALDI-TOF MS** | **Data limitations** |
| --- | --- | --- | --- | --- | --- |
| 1 | Karlstad central hospital, Värmland | Included | 37 | 2013 |  |
| 2 | Falu hospital, Dalarna | Included | 22 | Before 2014 | Data from 2014-01-20. |
| 3 | Gävle hospital, Gävleborg | Included | 26 | 2014 |  |
| 4 | Karolinska University hospital laboratory, Stockholm | Included | 122 | Information not available. All isolates listed as identified by MALDI-TOF MS. |  |
| 5 | Central hospital Växjö, Kronoberg | Included | 43 | 2011 |  |
| 6 | Halmstad hospital, Halland | Included | 26 | Before 2016 | Data from 2016-02-06. |
| 7 | Kalmar county hospital, Kalmar | Included | 30 | 2012 |  |
| 8 | Jönköping Ryhov county hospital, Jönköping | Included | 38 | Before 2012 | Data from 2012-04-01. Risk of incomplete data due to system limitations. |
| 9 | Sundsvall region hospital, Västernorrland | Included | 27 | 2012 |  |
| 10 | Northern Älvsborg county hospital, Västra götaland | Included | 27 | 2016 |  |
| 11 | Southern Älvsborg hospital, Västra götaland | Included | 21 | 2011 | Risk of incomplete data until 2015-06-01 due to system limitations. |
| 12 | Sahlgrenska university hospital, Västra götaland | Included | 81 | 2016 |  |
| 13 | University Hospital of Umeå, Västerbotten | Included | 23 | 2013 |  |
| 14 | Clinical microbiology, Skåne | Included | 254 | 2011 |  |
| 15 | Sunderby hospital, Norrbotten | Included | 30 | 2015 | Data from 2012-01-01. |
| 16 | Visby hospital, Gotland | Included | 8 | 2018 |  |
| 17 | Västerås central hospital, Västmanland | Included | 40 | 2015 |  |
| 18 | Unilabs:  Skaraborg, Västra götaland;  Eskilstuna, Södermanland;  St. Göran hospital, Stockholm | Included | 75  (Skaraborg 35, Eskilstuna 11, St. Göran – Stockholm 29) | Skaraborg 2011, Eskilstuna 2012, St. Göran –Stockholm 2012 |  |
| 19 | Uppsala university hospital, Uppsala | Declined |  |  |  |
| 20 | Linköping univeristy hospital, Östergötland | Declined |  |  |  |
| 21 | Örebro university hospital, Örebro | Excluded due to prolonged wait time |  |  |  |
| 22 | Östersund hospital, Jämtland | Excluded due to prolonged wait time |  |  |  |
| 23 | SYNLAB Stockholm | Excluded due to limited hospital size, no SBSEC episodes expected |  |  |  |
|  | **Total** | **18 included.**  **5 excluded.** | **930** |  |  |

**Table S1. Clinical microbiology laboratories in Sweden, data coverage.**

| **The National Patient Registry – ICD10** | |
| --- | --- |
| Benign colorectal tumours | K621, K635, D120, D122, D123, D124, D125, D126, D127, D128 |
| Infective endocarditis | I33, I389, I398 |
| Myocardial infarction^a)^ | I21, I22, I252 |
| Congestive heart failure^a)^ | I110, I130, I132, I255, I420, I426, I427, I428, I429, I43, I50 |
| Peripheral vascular disease^a)^ | I70, I71, I731, I738, I739, I771, I790, I792, K55 |
| Cerebrovascular disease^a)^ | G45, I60, I61, I62, I63, I64, I67, I69 |
| Chronic obstructive pulmonary disease^a)^ | J43, J44 |
| Other chronic pulmonary disease^a)^ | J41, J42, J45, J46, J47, J60, J61, J62, J63, J64, J65, J66, J67, J68, J69, J70 |
| Rheumatic disease^a)^ | M05, M06, M123, M070, M071, M072, M073, M08, M13, M30, M313, M314, M315, M316, M32, M33, M34, M350, M351, M353, M45, M46 |
| Dementia^a)^ | F00, F01, F02, F03, F051, G30, G311, G319 |
| Hemiplegia^a)^ | G114, G80, G81, G82, G830, G831, G832, G833, G838 |
| Diabetes without chronic complication^a)^ | E100, E101, E110, E111, E120, E121, E130, E131, E140, E141 |
| Diabetes with chronic complication^a)^ | E102, E103, E104, E105, E107, E112, E113, E114, E115, E116, E117, E122, E123, E124, E125, E126, E127, E132, E133, E134, E135, E136, E137, E142, E143, E144, E145, E146, E147 |
| Renal disease^a)^ | I120, I131, N032, N033, N034, N035, N036, N037, N052, N053, N054, N055, N056, N057, N11, N18, N19, N250, Q611, Q612, Q613, Q614, Z49, Z940, Z992 |
| Liver disease^a)^ | B15, B16, B17, B18, B19, K703, K709, K73, K746, K754 R18 I850, I859, I982, I983 |
| Peptic ulcer disease^a)^ | K25, K26, K27, K28 |
| Malignancy^a)^ | C00, C01, C02, C03, C04, C05, C06, C07, C08, C09, C10, C11, C12, C13, C14, C15, C16, C17, C18, C19, C20, C21, C22, C23, C24, C25, C26, C27, C28, C29, C30, C31, C32, C33, C34, C35, C36, C37, C38, C39, C40, C41, C42, C43, C44, C45, C46, C47, C48, C49, C50, C51, C52, C53, C54, C55, C56, C57, C58, C59, C60, C61, C62, C63, C64, C65, C66, C67, C68, C69, C70, C71, C72, C73, C74, C75, C76, C81, C82, C83, C84, C85, C86, C88, C89, C90, C91, C92, C93, C94, C95, C96, C97 |
| Metastatic cancer^a)^ | C77, C78, C79, C80 |
| Aids^a)^ | B20, B21, B22, B23, B24, F024, O987, R75, Z114, Z219, Z717 |
| **The National Prescribed Drug Register - ATC** | |
| Diabetes mellitus medications | A10 |
| Inhalation medication for chronic pulmonary disease | R03 |

**Table S2. Registered codes in The National Patient Registry and The National Prescribed Drug Register.** ^a)^Included in Cox regression analysis using entropy balancing and calculation of the Charlson comorbidity index score.

| **Colorectal cancer diagnosed during follow-up of the colorectal cancer cohort in different subgroups** | | **Exposed – SBSEC** | **Unexposed controls** | *P* |
| --- | --- | --- | --- | --- |
| ***Streptococcus gallolyticus* species^a)^** | | **n = 259** | **n = 2542** |  |
| Total | Colorectal cancer, *n* | 17 | 26 |  |
|  | Incidence per  1000 person-years | 18.5 | 2.0 | <0.01 |
|  | Incidence difference (CI 95%) | 16.5 (7.7 – 25.3) | | |
| Year <1 | Colorectal cancer, *n* | 12 | 4 |  |
|  | Incidence per  1000 person-years | 56.9 | 1.6 | <0.01 |
|  | Incidence difference (CI 95%) | 55.2 (23.0 – 87.5) | | |
| ≥1 year | Colorectal cancer, *n* | 5 | 22 |  |
|  | Incidence per  1000 person-years | 7.0 | 2.1 | 0.03 |
|  | Incidence difference (CI 95%) | 5.0 (-1.3 – 11.2) | | |
| **Other defined SBSEC species^b)^** | | **n = 111** | **n = 1078** |  |
| Total | Colorectal cancer, *n* | 5 | 12 |  |
|  | Incidence per  1000 person-years | 19.0 | 2.4 | <0.01 |
|  | Incidence difference (CI 95%) | 16.5 (-0.1 – 33.2) | | |
| <1 year | Colorectal cancer, *n* | 3 | 4 |  |
|  | Incidence per  1000 person-years | 34.8 | 3.8 | <0.01 |
|  | Incidence difference (CI 95%) | 31.0 (-8.6 – 70.6) | | |
| ≥1 year | Colorectal cancer, *n* | 2 | 8 |  |
|  | Incidence per  1000 person-years | 11.2 | 2.0 | 0.08 |
|  | Incidence difference (CI 95%) | 9.2 (-6.4 – 24.8) | | |
| **Infectious endocarditis** | | **n = 96** | **n = 941** |  |
| Total | Colorectal cancer, *n* | 12 | 8 |  |
|  | Incidence per  1000 person-years | 38.2 | 1.7 | <0.01 |
|  | Incidence difference (CI 95%) | 36.5 (14.9 – 58.2) | | |
| <1 year | Colorectal cancer, *n* | 8 | 2 |  |
|  | Incidence per  1000 person-years | 105.1 | 2.2 | <0.01 |
|  | Incidence difference (CI 95%) | 102.9 (30.4 – 175.8) | | |
| ≥1 year | Colorectal cancer, *n* | 4 | 6 |  |
|  | Incidence per  1000 person-years | 16.8 | 1.6 | <0.01 |
|  | Incidence difference (CI 95%) | 15.2 (-0.1 – 31.8) | | |
| **No infectious endocarditis** | | **n = 635** | **n = 6217** |  |
| Total | Colorectal cancer, *n* | 33 | 81 |  |
|  | Incidence per  1000 person-years | 17.4 | 2.8 | <0.01 |
|  | Incidence difference (CI 95%) | 14.6 (8.6 – 20.6) | | |
| <1 year | Colorectal cancer, *n* | 27 | 18 |  |
|  | Incidence per  1000 person-years | 52.7 | 3.0 | <0.01 |
|  | Incidence difference (CI 95%) | 49.7 (29.8 – 69.7) | | |
| ≥1 year | Colorectal cancer, *n* | 6 | 63 |  |
|  | Incidence per  1000 person-years | 4.3 | 2.7 | 0.29 |
|  | Incidence difference (CI 95%) | 1.6 (-1.9 – 5.1) | | |

**Table S3. Colorectal cancer diagnosed during follow-up of the colorectal cancer cohort for different subgroups.** ^a)^ Includes *Streptococcus gallolyticus* subsp. *gallolyticus*, *Streptococcus gallolyticus* subsp. *pasteurianus*, and *Streptococcus gallolyticus* subsp. *macedonicus*. ^b)^ Includes *Streptococcus* *lutetiensis* (previously *Streptococcus* *infantarius* subsp. *coli*), *Streptococcus* *infantarius* (previously *Streptococcus* *infantarius* subsp. *infantarius*), *Streptococcus* *equinus*, and *Streptococcus* *alactolyticus*.

| **Stratification by matching groups** | **Hazard ratio (95 % confidence interval)** |
| --- | --- |
| SBSEC relapse episodes included | 10.0 (6.6 – 15.4) |
| Only controls with previous hospital stay with a diagnosis included in Charlson comorbidity index | 9.8 (3.8 – 24.9) |
| Only SBSEC and controls with previous hospital stay with a diagnosis included in Charlson comorbidity index | 9.5 (4.8 – 19.0) |
| **Entropy balancing^a)^** | **Hazard ratio (95 % confidence interval)** |
| SBSEC relapse episodes included | 6.7 (4.3 – 10.4) |
| Only controls with previous hospital stay with a diagnosis included in Charlson comorbidity index | 5.6 (2.9 – 10.9) |
| Only SBSEC and controls with previous hospital stay with a diagnosis included in Charlson comorbidity index | 5.7 (2.9 – 11.4) |
| Rebalanced weights after exclusion of weights in the 99^th^ percentile. | 7.7 (4.9 – 12.1) |
| All controls included^b)^ | 6.9 (4.5 – 10.7) |

**Table S4. Sensitivity analyses of colorectal cancer diagnosed during follow-up.** ^a)^Entropy balancing using the index year, geographic region, sex, age, and comorbidities. ^b)^Controls of the follow-up of the colorectal cancer cohort used in other analyses.
